# Genetic Dissection of Epistatic Interactions Contributing Yield-Related Agronomic Traits in Rice Using the Compressed Mixed Model

**DOI:** 10.3390/plants11192504

**Published:** 2022-09-26

**Authors:** Ling Li, Xinyi Wu, Juncong Chen, Shengmeng Wang, Yuxuan Wan, Hanbing Ji, Yangjun Wen, Jin Zhang

**Affiliations:** 1 College of Science, Nanjing Agricultural University, Nanjing 210095, China; 2 College of Finance, Nanjing Agricultural University, Nanjing 210095, China; 3 School of Business Administration, Jiangxi University of Finance and Economics, Nanchang 330013, China; 4 Key Laboratory of Crop Genetics and Germplasm Enhancement, Nanjing Agricultural University, Nanjing 210095, China

**Keywords:** GWAS, QTN-by-QTL interaction, epistatics, yield-related traits, rice

## Abstract

Rice (*Oryza sativa*) is one of the most important cereal crops in the world, and yield-related agronomic traits, including plant height (PH), panicle length (PL), and protein content (PC), are prerequisites for attaining the desired yield and quality in breeding programs. Meanwhile, the main effects and epistatic effects of quantitative trait nucleotides (QTNs) are all important genetic components for yield-related quantitative traits. In this study, we conducted genome-wide association studies (GWAS) for 413 rice germplasm resources, with 36,901 single nucleotide polymorphisms (SNPs), to identify QTNs, QTN-by-QTN interaction (QQI), and their candidate genes, using a multi-locus compressed variance component mixed model, 3VmrMLM. As a result, two significant QTNs and 56 paired QQIs were detected, amongst 5219 genes of these QTNs, and 26 genes were identified as the yield-related confirmed genes, such as LCRN1, OsSPL3, and OsVOZ1 for PH, and LOG and QsBZR1 for PL. To reveal the substantial contributions related to the variation of yield-related agronomic traits in rice, we further implemented an enrichment analysis and expression analysis. As the results showed, 114 genes, nearly all significant QQIs, were involved in 37 GO terms; for example, the macromolecule metabolic process (GO:0043170), intracellular part (GO:0044424), and binding (GO:0005488). It was revealed that most of the QQIs and the candidate genes were significantly involved in the biological process, molecular function, and cellular component of the target traits. The demonstrated genetic interactions play a critical role in yield-related agronomic traits of rice, and such epistatic interactions contributed to large portions of the missing heritability in GWAS. These results help us to understand the genetic basis underlying the inheritance of the three yield-related agronomic traits and provide implications for rice improvement.

## 1. Introduction

Rice (*Oryza sativa*) is one of the most important cereal crops in the world. It currently contributes to the daily energy needs of half of the world’s population [1], especially in Asia. Increasing grain yields is a long-term goal in rice breeding, to meet the demands of global food security. The important yield-related agronomic quantitative traits, including plant height (PH), panicle length (PL), and grain protein content (PC), are highly correlated with yield and quality in rice [2,3,4]. Meanwhile, appropriate yield-related agronomic traits influence rice status and yield potential; therefore, they are pre-requisites for attaining the desired yield and quality targets. It is essential to dissect the yield-inherited traits, mine the novel associated genes, and elucidate the genetic bases. In the past decades, a large number of studies have provided strong support for the genetic improvement of rice yield-inherited traits [2,3,4,5]. However, several associated genes remain unexplored and cause a “missing heritability” problem. It has been hypothesized that multivariate analysis and the detection of epistatic interactions between markers may help resolve the above problem, currently observed in genome-wide association studies (GWAS).

Epistasis, non-allelic interactions, is one of the important genetic factors making a substantial contribution to the variation in complex traits [6], such as grain yield. It was previously described as an intergenic reciprocal phenomenon, in which the phenotypic effect of one locus overshadows the phenotypic effect of another locus [7]. Recent studies have indicated that epistasis plays an important role in the genetic dissection of the quantitative traits [8] of plants. The recent advances in molecular marker technology and developing analytical methods based on high-density molecular markers, to identify epistasis efficiently, are critical to understanding the genetic basis [9,10] and have attracted a wide range of research [11].

To date, a number of methods and software packages have been proposed to detect quantitative trait loci (QTN) main effects and epistatic effects, namely QTN-by-QTN interactions (QQIs). PLINK [12,13] offers a simple but potentially powerful approach to process genome-wide association analysis data. SNPHarvester [14], FastEpistasis [15], and BOOST [16] serve as efficient tools for interaction mapping in GWAS. These algorithms have a lower computational volume and computational complexity, at the expense of their accuracy [10]. Machine learning is an alternative method to dissect the interactions in genetic analysis, such as multifactor dimensionality reduction [17] and random forest algorithms [18,19]. These approaches aim to select a subset of (single nucleotide polymorphisms) SNPs for interaction tests, on the basis of existing biological knowledge (gene based [10]) or statistical features (marginal effects [20,21]), and methods based on variance heterogeneity among SNP genotypes, which could miss SNPs that are interacting but that have limited variance heterogeneity. Other methodologies have also been developed for detecting epistasis [11].

From the perspective of controlling population structure and polygenic background, and improving computational accuracy, the mixed linear model (MLM) method for GWAS has been established [22,23,24], and a series of algorithms have been proposed [25,26,27]. However, several potentially associated loci cannot pass the stringent threshold of Bonferroni correction; thus, the single-locus methods fail to capture the potentially important loci of complex traits, especially for the large experimental errors inherent in field experiments of crop genetics [28]. To address this issue, multi-locus GWAS methodologies have been recommended, such as mrMLM [29], pLARmEB [30], and FASTmrEMMA [31,32]. These multi-locus GWAS methodologies show their advantages in terms of QTN detection power and effect estimation accuracy, even under the circumstance where the number of loci is greater larger than the sample size [33,34].

All the above single- and multi-locus methods under the framework of MLM are based on the main effect model, the epistatic interaction of alleles between different loci is not considered, which is thought to play a crucial role in quantitative trait genetic analysis and is suggested as a key reason for ascribing missing heritability as well. However, there are two main challenges to discovering epistasis: computational complexity, and statistical power [10,35]. For the first challenge, the number of interactions exponentially increases along with more loci being considered; the estimation of variance components is computationally expensive under the large-scale loci of MLM. Second, since a huge number of statistical tests and effect estimations are conducted on a limited sample size, with interactions of an exhaustive search, false positives and overfitting might arise.

In order to efficiently identify more epistatic interactions with both statistical and biological significance, a multi-locus linear mixed model with compressed variance components was proposed, named 3VmrMLM [9], which combines the merits of the multi-locus approach in identifying more associated loci and the accuracy of effects estimation at a satisfactory computing speed. 3VmrMLM first estimates the genotype effects, and subsequently uses an analysis of variance (ANOVA) model to divide the estimated genotype effects. It effectively reduces the computational complexity by compressing the number of variance components, from fifteen to three, in the epistatic model. The 3VmrMLM method has efficiently detected potentially associated loci and almost unbiasedly estimated their effects, with high statistical powers and accuracies, and a low false positive rate. It provides a novel approach to revealing the genetic basis of quantitative traits [9].

In the last two decades, there have been several reported epistatic interactions of yield-related traits in rice. Xing et al. [36] detected a total of 35 interactions for yield traits in RIL. Liu et al. [37] estimated the additive, dominant, and dominant-dominant epistatic effects for yield-related traits. Stable epistatic QTL identification significantly improved molecularly designed breeding [38]. The epistatic effects for yield were identified in brewing rice [39]. The identification of these epistatic interactions based on single-locus analyses provides valuable resources for gene discovery and yield improvement.

However, yield-related agronomic traits are usually controlled by various polygenes and epistatic interactions; thus, they are undetectable for analysis by the single-locus methods. Therefore, we should apply a multi-locus linear mixed model to identify loci and epistatic interactions related to yield-related quantitative traits. In this study, 413 rice germplasm resources with 44 K SNPs and three quantitative traits (plant height, panicle length, and grain protein content) were analyzed using the 3VmrMLM method. We identified the yield-related agronomic QTNs and QQIs in rice, and mined candidate genes in the neighborhood of the significant QTNs and QQIs. In addition, we performed a pathway enrichment analysis and tissue expression analysis, which helped us to understand the genetic bases underlying the inheritance of the three traits, and provided implications for rice improvement.

## 2. Materials and Methods

### 2.1. Rice Datasets

To identify the significant genomic loci and interactions associated with yield-related agronomic traits in rice, 413 rice germplasm resources from the Rice Diversity Database (https://www.ricedata.cn/gene/ (accessed on 19 May 2022)) were used in this study for GWAS. The dataset consists of 413 inbred accessions with high-quality 44,100 SNPs from 82 countries. After filtering, 36,901 SNPs with minor allele frequency (MAF) >0.01 were analyzed in this study. The phenotypic datasets are available from the Rice Diversity website (https://www.ricedata.cn/ (accessed on 19 May 2022)). To gain insight into the three yield-related agronomic traits, we implemented descriptive statistics for phenotypic data using R software (https://www.r-project.org/ (accessed on 18 June 2022)), the results of descriptive statistics included the mean, standard deviation (SD), coefficient of variation (CV), minimum, maximum, and range for the three traits.

### 2.2. Dimensionality Reduction

Considering all interactions between each SNP in the model, the calculation burden will increase dramatically. For example, the number of SNPs is 36,901, and the interactions are over a half billion. To avoid an exhaustive epistatic interaction search and decrease the computational volume and computational complexity, two techniques were adopted in this study for reducing the dimensionality of SNPs: linkage disequilibrium (LD) analysis, and linear model (LM) regression analysis. LD is a non-random association between alleles at different loci, which does exist for many pairs of SNPs. It is estimated as the square of the correlation coefficient of the paired alleles. More studies are subject to an inflated level of false-positive results when the two SNPs are highly correlated and/or both have significant marginal effects [11,40], it would be better to filter them out using LD analysis. For each pair of SNPs, LD can be tested using the PLINK toolset. The command for LD analysis is “*plink* --*file* rice --*pheno* phenotype.txt --*assoc* --*out* rice_ld --*allow-no-sex*”. The threshold for LD analysis is 0.8 [41]. If the number of SNP variables is greater than five thousand (the recommended number for epistatic interactions analysis in 3VmrMLM), single-locus LM analysis is recommended [23]. The *p*-value is calculated using the t-test of PLINK. The command for LM analysis is “*plink* --*file* rice_ld --*pheno* phenotype.txt --*allow-no-sex* --*linear* --*out* rice_res”. Bonferroni correction is employed as the significance criterion.

### 2.3. Genome-Wide Association Study

SNP filtering after the dimensionality reduction was used to construct the multi-locus model for the 3VmrMLM method. There are fifteen variance components in the QQI (epistatic interactions) mixed model [9], and this greatly increases the computing burden. After recoding the genotype and the combination of polygenetic backgrounds, the number of variance components was decreased from fifteen to three in the model; for more details see Li et al. [9]. Thus, the 3VmrMLM method estimates the combined effect of a pair of QTNs and then divides them into *a_t_* and *d_t_* for *t*th SNP, *a_s_* and *d_s_* for *s*th SNP, and additive-by-additive (*aa*), additive-by-dominance (*ad*), dominance-by-additive (*da*), and dominance-by-dominance (*dd*) interaction effects using a two-way ANOVA model. All the effects are estimated by EM empirical Bayes [42]. The threshold *p*-value < 5.00 × 10^−8^ or LOD ≥ 3.00 in genome-wide detection is applied [31,43].

The “*IIIVmrMLM*” package was downloaded from https://github.com/YuanmingZhang65/IIIVmrMLM (accessed on 26 June 2022). We conducted main-effect QTN detection and QTN-by-QTN detection, both using the “*IIIVmrMLM*” function, specifying the parameter of “*method* = c(“Single_env”)” for the main-effect QTN detection model and “*method* = c (“Epistasis”)” for the QTN-by-QTN detection model.

### 2.4. Candidate Gene Identification and Enrichment Analysis

The China Rice Data Center (CRDC) database (https://ricedata.cn/ (accessed on 3 July 2022)) was used to annotate the significant loci (main effect SNPs and epistatic interactions) identified by 3VmrMLM in this study. The regions within 200 kb of all significant loci were used to search for the candidate genes.

To further understand the genetic basis, we performed gene ontology (GO) enrichment analysis, based on the nearest candidate genes at significant loci, which provides more information on biological functions, pathways, or cellular localizations [44]. The online tool agriGO (http://systemsbiology.cau.edu.cn/agriGOv2/# (accessed on 25 July 2022)) [45] was used to perform a GO enrichment analysis concerning biological process (BP), molecular function (MF), and cellular component (CC) for the candidate genes, to identify the genes that may be significantly associated with the yield-related agronomic traits. To summarize, GO enrichment analysis was conducted using a single enrichment analysis tool, and Fisher’s exact test (*p*-value < 0.05) was utilized to select enrichment GO terms. The R package “*pheatmap*” was used to plot the heatmap according to the results of the GO analysis for the candidate genes.

### 2.5. Tissue-Specific Expression Analysis

The Rice Genome Annotation Project (RGAP) database (http://rice.uga.edu/ (accessed on 3 August 2022)) was adopted to show the expression levels of the candidate genes in various tissues or organs, and we used the R package “*pheatmap*” to draw the heat map, which illustrates the fragments per kilobase of exon model per million mapped fragments (FPKM) expression values of the candidate genes through the tissues or organs.

## 3. Results

### 3.1. Phenotypic Variation 

**Figure 1:**
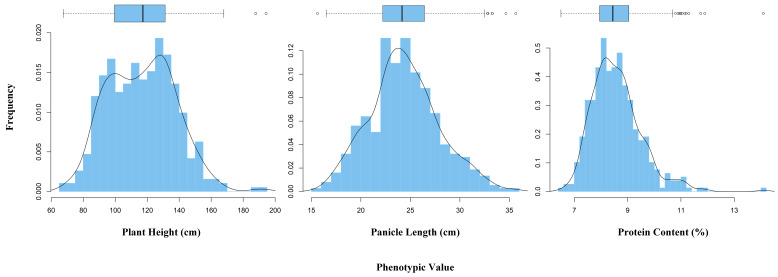
Descriptive statistics of phenotypic values for the three yield-related traits (PH, PL, and PC).

Three yield-related agronomic traits (including PH, PL, and PC) were analyzed, to examine whether there were significant phenotypic variations among the 413 rice varieties. Figure 1 shows the variations in phenotypic values for the three traits using a distribution profile, box plot, and histogram. According to the Kolmogorov–Smirnov test of the three traits, all the phenotypes approximately follow the normal distribution. In addition, the means of PH, PL, and PC were 116.58 cm, 24.37 cm, and 8.59%, respectively (Supplementary Table S1). Obviously, the phenotypic datasets of PH, Pl, and PC are widely spread: from 67.95 to 194.33, from 15.63 to 35.68, and from 6.50 to 14.10, respectively. Meanwhile, the trait of PC had the minimum variance (Figure 1, Supplementary Table S1), this means the phenotypic datasets of PC were concentrated around the mean value. Both the SD (21.09) and CV (0.18) of PH are greater than that of PL (SD: 3.54, CV: 0.15) and PC (SD: 0.94, CV: 0.11) (Supplementary Table S1). The detailed descriptive statistics, including the mean, SD, CV, minimum, maximum, and range for the three phenotypes are presented in Supplementary Table S1.

### 3.2. Genetic Dissection of Epistatic Interactions

All the SNPs were analyzed using a dimensionality reduction step, and 4078 and 4000 SNPs were used to construct the multi-locus model of the 3VmrMLM GWAS method for PH, PL, and PC, respectively. A total of two significant QTNs and 56 paired QQIs (Supplementary Table S2, Supplementary Figure S1) were detected for the three traits using the 3VmrMLM method (LOD ≥ 3.00 or *p* value < 5.00 × 10^−8^). The total QTNs and QQIs explained 59.85%, 31.97%, and 67.36% of the phenotypic variations (phenotypic variation explained, PVE values), which were calculated as the proportion of the variance of all the QTNs to the variance of each phenotype using the “*IIIVmrMLM*” R package. For PH 1 QTN and 14 paired QQIs were found to be tightly associated with the target trait, which were widely located on all 12 chromosomes, except chromosome 10. The PVE of QTN was 12.83%, with a LOD score of 6.79, while the PVE of QQIs ranged from 1.14% to 7.15% with the LOD score from 3.23 to 8.16 (Table 1, Supplementary Figure S1), and the total PVE of QQIs was 47.02%. This means epistatic interactions made a substantial contribution related to the variation of yield-related agronomic traits in rice, which is consistent with Yu et al. [46]. Among all QQIs, marker id2001400 located at 2,233,430 bp on chromosome 2, along with the maximum value of PVE 7.15%, were validated as associated with the yield-related traits. There were 21 paired QQIs associated with PL trait identified, which were distributed on chromosomes 1–8, 10, and 11. The PVE of QQIs ranged from 0.03% to 4.41%, with the LOD score from 3.38 to 9.09 (Supplementary Table S2, Supplementary Figure S1). In addition, 1 QTN and 21 paired QQIs spread over chromosomes 1–8 and 10–12 were related to PC. The PVE of QTN was 5.20%, with a LOD score of 4.02; while the PVE of QQIs ranged from 0.30% to 10.04%, with the LOD score from 3.04 to 9.07 (Supplementary Table S2, Supplementary Figure S1); the total PVE of QQIs was 62.16%. The genetic dissection demonstrated that epistasis plays an important role in the genetic dissection of all three yield-related agronomic traits.

We compared the confirmed genes with all significant QTNs or QQIs and their genomic ranges (200 kb up- and down-stream around the associated QTNs) by https://www.ricedata.cn/gene/. For PH, 11 paired QQIs (Table 1, Supplementary Table S2); that is, over two-thirds of QQIs were adjacent to or overlapped with the confirmed genes, which were demonstrated to be associated with the yield-related traits. Interestingly, we dissected several confirmed clusters of genes for the target trait; for example, the locus id6001114, which was located at 1,524,748bp on chromosome 6, overlapped with four confirmed genes of PH, LOC_Os06g03710 (DLT; SMOS2), LOC_Os06g03770 (OsATM3), LOC_Os06g03810 (ROD1), and LOC_Os06g04010 (OsGBP1). Meanwhile, marker id5013209 on chromosome 5 overlapped with OsPDCD5, a gene known to be involved in cell death, which has recently been demonstrated to negatively regulate the plant and grain yield of rice by Dong et al. [47]. According to the experiments, the OsPDCD5 gene was verified as a powerful candidate gene for high-yield and quality rice. For PL and PC, five and four paired QQIs (Supplementary Table S2) were adjacent to, or overlapped with, the confirmed genes, respectively. From the perspective of the confirmed genes, epistasis interactions contributed to the large portions of the missing heritability of all three yield-related agronomic traits in GWAS.

### 3.3. Functional Enrichment Analysis of the Candidate Genes 

**Figure 2:**
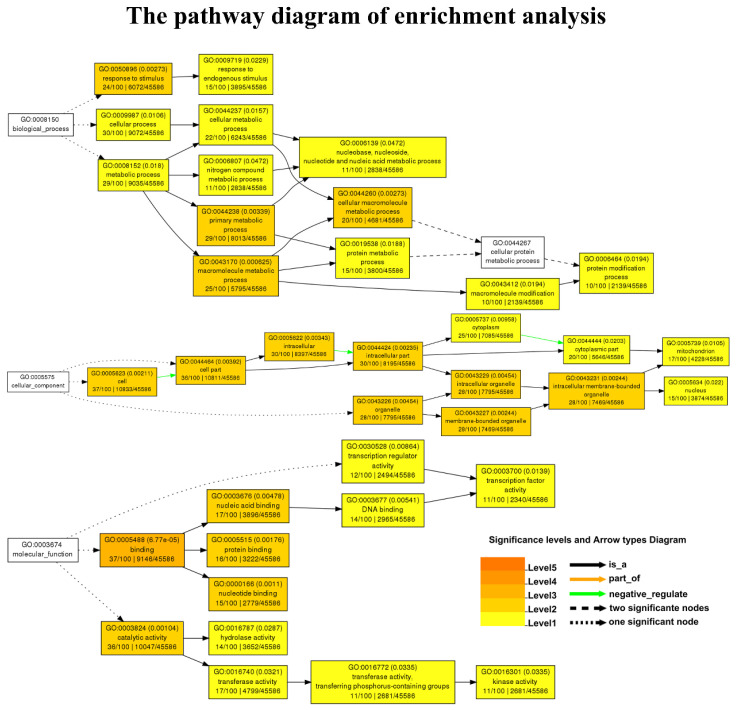
Annotated hierarchical tree of GO (slim) annotations for predicted SNP markers. It consists of three categories: biological process (BP), molecular function (MF), and cellular component (CC). The boxes in the figure represent GO terms, consisting of their correspondence to the ID inside the GO database, the GO term functional description, and the number of differential genes enriched to that term. Significant (*p* ≤ 0.05) and nonsignificant terms are labeled with colored and white boxes, respectively, and the color in the each box reflects the enrichment of differential genes in the GO term; the darker color, the more significant the enrichment. The left and right ends of the arrows represent the hierarchical relationship between the upper and lower levels of the GO term, with the arrow pointing to a lower level.

GO analysis is a powerful bioinformatics tool for better understanding the underlying BP of candidate genes, as well as their MF and CC. Consequently, to gain an insight into the genetic basis of the candidate genes, we conducted GO enrichment analysis, the results of which are presented in Figure 2 and Supplementary Figure S1. According to the outcomes of the GO functional enrichment study, 114 candidate genes of QTNs and QQIs were significantly (*p*-value < 0.05) enriched for 37 GO terms associated with various BPs (Figure 2). In the rectangular boxes of Figure 2, the most significant pathways or GO terms containing the candidate genes are marked in a darker color. One term, GO: 0043231, was involved in the CCs with intracellular membrane-bounded organelles. The MF mainly described activities that occurred at the molecular level, and one of the significant nodes was GO: 0030528, which was involved in transcription regulator activity. Among the identified BPs, the primary metabolic process (GO:0044238) is one of the crucial pathways that is functionally linked to both metabolic processes (GO:0008152) and cellular processes (GO:0009987) in rice. The metabolic pathway performs metabolic activities, to convert food into energy and run cellular processes that form proteins, lipids, nucleic acids, and some carbohydrate components. In the last layer (Figure 2), 10 genes were enriched to the protein modification process (GO: 0006464), and the newly identified genes LOC_Os01g02300, LOC_Os01g51400, LOC_Os06g48530, LOC_Os11g41820, and LOC_Os12g14610 were involved in this process (Figure 2 and Supplementary Figure S1), allowing rice plants to grow, reproduce, and maintain their structure. This information may help to provide a biological basis for the newly discovered genes and help to identify yield-related genes in rice.

### 3.4. Expression Profile of the Candidate Genes 

**Figure 3:**
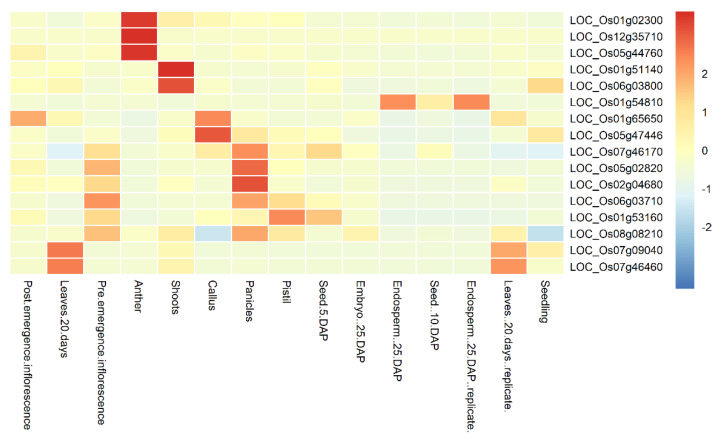
Heatmap showing FPKM values of a subset of candidate genes for the identified PH traits. The heatmap reflects the expression in different organs or tissues of rice.

The expression levels of candidate genes in different organs or tissues, including shoots, anther, seeds, leaves, panicles, endosperm, and inflorescence can be queried in the database RGAP. Figure 3 exhibits the FPKM expression values of candidate genes for the PH trait across various tissues or organs (normalized before heat map plotting). The corresponding results for the remaining traits are shown in Supplemental Table S3. As can be seen from Figure 3, gene LOC_Os 01g54810 had the highest FPKM expression value in both Endospem 25 DAP and Endospem 25 DAP replicates. Gene LOC_Os 07g46460 had the highest level of expression in rice 20-day leaves. The genes LOC_Os 07g46170, LOC_Os 05g02820, LOC_Os 02g04680, LOC_Os 06g03710, and LOC_Os 08g08210 showed higher expressions in panicle and preemergence inflorescence. The genes LOC_Os 01g02300, LOC_Os 12g35710, and LOC_Os 0544760 showed high expressions in anthers. The important effect of anthers and panicles in regulating rice yield has been shown in previous studies [48,49].

## 4. Discussion

In this study, a novel method 3VmrMLM was adopted to identify significant QTNs and QQIs in three yield-related agronomic traits of 413 rice germplasm resources, and 58 significant QTNs and QQIs were detected (Table 1, Supplementary Figure S1 and Supplemental Table S2). Among the three traits, the maximum quantity of QTNs and QQIs corresponding to confirmed genes were identified for the PH trait, where over two-thirds of these loci also overlapped or were adjacent to the confirmed genes. Additionally, 21 paired QQIs corresponding to five confirmed genes were associated with PL: one QTN and 21 paired QQIs corresponding to four confirmed genes associated with PC were simultaneously detected by 3VmrMLM.

To further validate the epistatic interaction results of the three yield-related agronomic traits by 3VmrMLM, we compared the single-locus epistasis detection method with a marginal epistasis test, the rapid epistatic mixed model association analysis method (REMMA) [50]. The main idea of REMMA is to apply an extended genomic best linear unbiased prediction model (EG-BLUP) and additive and non-additive kinship matrices to perform estimation, and then linearly re-transform the estimated effects, to obtain the epistatic interaction effects. In this study, 413 rice accessions with 36,901 SNPs were re-analyzed to identify QTNs and QQIs for yield-related agronomic traits using REMMA. The results showed that ~2,250,000 significant QTNs and QQIs after Bonferroni correction were simultaneously detected as associated with PH traits, and the model over fit the epistasis tests and did not generate any meaningful results. Meanwhile, no significant loci were detected after Bonferroni correction for the PL and PC traits using REMMA. All the results imply that 3VmrMLM is a stable and powerful method under different genetic backgrounds.

3VmrMLM is a powerful multi-locus tool to identify the main effects QTNs and epistatic interaction effects QQIs, simultaneously estimate and test their genetic effects, and so on. As an advanced approach, its merits can be explained as the following points: (1) 3VmrMLM, based on a multi-locus approach, adds the polygenic effect and population structure, to decrease the bias in effect estimations by controlling the genetic background, and it might be relatively close to the true genetic models of plants and animals, thus 3VmrMLM produces high-quality results, with higher statistical power and lower false positive rate (FPR). (2) To reduce the computational complexity, 3VmrMLM employs the two stages: first, all putative QTNs are chosen by a single-locus model, then the QTNs after filtering are being included in a multi-locus model for true QTN detection. This is the key point for 3VmrMLM to efficiently solve the “big P, small N” problem (larger-scale markers or interactions). (3) In the QQI detection model, the number of variance components is decreased from fifteen to three, and this is one of the most obvious advantages for solving the problem of the huge computational resources needed by too many markers. (4) For the single-locus method, the multiple test correction of the significance test (Bonferroni correction) was too stringent to capture all true QTNs. Differently from this, 3VmrMLM first uses single marker genome-wide scans to select potentially relevant markers and then uses empirical Bayes and likelihood ratio tests to detect the above loci in a multi-locus model. The appropriate threshold and polygenic model are the desirable features of 3VmrMLM [9], thus it provides more theoretical support for crop improvement.

## 5. Conclusions

This study conducted a GWAS for yield-related agronomic traits in rice, including PH, PL, and PC. As a result, two significant QTNs and 56 paired QQIs were detected by 3VmrMLM, while REMMA failed to identify significant QQIs. For the QTNs and QQIs, 26 key genes were identified as confirmed yield-related genes, such as LCRN1, OsSPL3, and OsVOZ1 for PH; and LOG and QsBZR1 for PL. Subsequently, an enrichment analysis and expression analysis indicated that most of the 114 candidate genes were significantly involved in all three GO terms of the target traits. These results help us to understand the genetic bases underlying the inheritance of yield-related agronomic traits and provide implications for rice improvement.

## Figures and Tables

**Table 1:** Results for the significant QQIs of the trait PH using 3VmrMLM.

NO.	QTN1				QTN2											
	Chr	Pos	Gene ID	Gene	Chr	Pos	Gene ID	Gene	LOD	aa.Effect	ad.Effect	da.Effect	dd.Effect	Variance	PVE (%)	*p*-Value
QTN1	1	38111539							6.79	12.63			−19.11	57.09	12.832	1.62 × 10^−7^
QQI 1	1	723562			11	25849860			5.75	3.76				12.94	2.910	2.66 × 10^−7^
QQI 2	1	29384858			2	2233430	LOC_Os02g04680	LCRN1; OsSPL3	5.68	2.69	6.03			31.82	7.151	2.09 × 10^−6^
QQI 3	1	29557152			12	15325876			6.87	5.75				24.38	5.480	1.85 × 10^−8^
QQI 4	1	30547272	LOC_Os01g53160	OFP3; OsOFP04	5	1015771			5.56	−4.94				21.98	4.940	4.24 × 10^−7^
QQI 5	1	31651011	LOC_Os01g54810 LOC_Os01g54930	THIS1 OsVOZ1	9	9425939			3.23	−4.19				6.25	1.406	1.14 × 10^−4^
QQI 6	1	39419765	LOC_Os01g68000	PLA2; LHD2	6	3636360			6.97	3.69		2.58		9.16	2.059	1.07 × 10^−7^
QQI 7	3	13773095	LOC_Os03g24220	VLN2	9	11706989			5.72	4.36				8.18	1.839	2.89 × 10^−7^
QQI 8	4	29851050			12	21716878			6.32	−4.51				20.16	4.531	6.89 × 10^−8^
QQI 9	5	25953209	LOC_Os05g44310 LOC_Os05g44760	OsSec18 OsHXK5	6	1524748	LOC_Os06g03710 LOC_Os06g03770 LOC_Os06g03810 LOC_Os06g04010	DLT; SMOS2 OsATM3 ROD1 OsGBP1	4.65	3.20				8.66	1.946	3.69 × 10^−6^
QQI 10	5	27196868	LOC_Os05g47446	OsPDCD5	8	4731022	LOC_Os08g08210	SDG701	4.06	3.99				7.59	1.707	1.52 × 10^−5^
QQI 11	5	28309324			7	26640298			4.18	−4.67	−3.22			14.83	3.334	6.67 × 10^−5^
QQI 12	6	29357275	LOC_Os06g48530	Du13	7	6016810			5.88	2.54				5.07	1.139	1.95 × 10^−7^
QQI 13	7	4724800			7	27550702	LOC_Os07g46460	Fd-GOGAT1	4.79	5.73	−1.32			22.64	5.090	1.61 × 10^−5^
QQI 14	8	51045			9	854638			8.16	4.86				15.52	3.488	8.76 × 10^−10^

Chr: chromosome, Pos: marker’s position (bp) on the genome, variance: the variance of each QTN or QQI, PVE (%): the proportion of total phenotypic variance explained by each QTN or QQI.

## Data Availability

Data recorded in the current study are available at www.ricediversity.org (accessed on 19 May 2022).

## Supplementary Materials

The following supporting information can be downloaded at: https://www.mdpi.com/article/10.3390/plants11192504/s1, Figure S1: Manhattan plot of the three target traits using 3VmrMLM. Besides the blue and green dots, the same color dots represent a pair of QTN-by-QTN interactions; Figure S2: Expression map of GO for the 116 candidate genes. Represents the expression map of the functional pathways viz., biological process (BP), molecular function (MF), and cellular component (CC) of the ten candidate genes; Table S1: statistical descriptive analysis of three rice yield-related agronomic traits; Table S2: results for significant QTNs and QQIs of PH, PL, and PC using 3VmrMLM. Table S3: The FPKM expression values of all candidate genes for three yield-related agronomic traits.

## Abbreviations

PH, plant height; PL, panicle length; PC, protein content; QTN, quantitative trait nucleotide; QQI, QTN by QTN interaction; GWAS, genome-wide association study; SNP, single nucleotide polymorphism; MLM, the mixed linear model; mrMLM, multi-locus random-SNP-effect mixed linear model; pLARmEB, polygenic-background-control-based least angle regression plus empirical Bayes; FASTmrEMMA, a fast multi-locus random-SNP-effect efficient mixed-model association; FASTmrMLM, a fast mrMLM multilocus mixed linear model; ANOVA, analysis of variance; QTL, quantitative trait locus; MAF, minor allele frequency; CV, coefficient of variation; LD, linkage disequilibrium; LM, linear model; EM, expectation and maximization; CRDC, China Rice Data Center; GO, gene ontology; BP, biological process, CC, cellular component, MF, molecular function; RGAP, the rice genome annotation project; FPKM, fragments per kilobase of exon model per million mapped fragments; LOD, limit of detection; PVE, phenotypic variation explained; REMMA, the rapid epistatic mixed model association analysis method; QEI, QTL-by-environment interaction; FPR, false positive rate.
