# Supplementary figures and images for: Genetic Dissection of Epistatic Interactions Contributing Yield-Related Agronomic Traits in Rice Using the Compressed Mixed Model
Source: Plants (Basel). 2022 Sep 26;11(19):2504. doi: 10.3390/plants11192504 (PMC9571936; doi:10.3390/plants11192504)

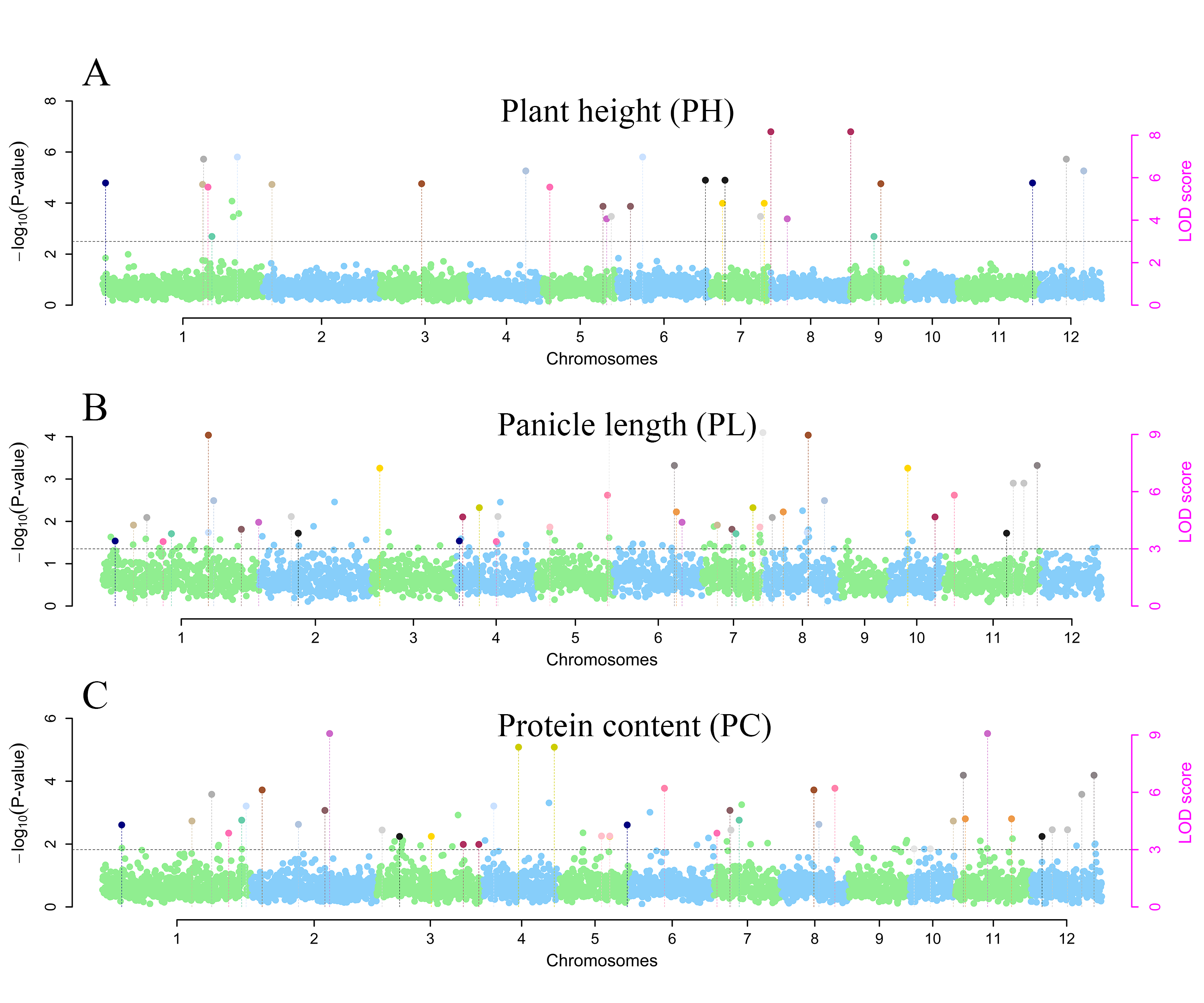

Supplement: Supplementary file 1 [file plants-11-02504-s001.zip › Supplementary Figure S1.tif]

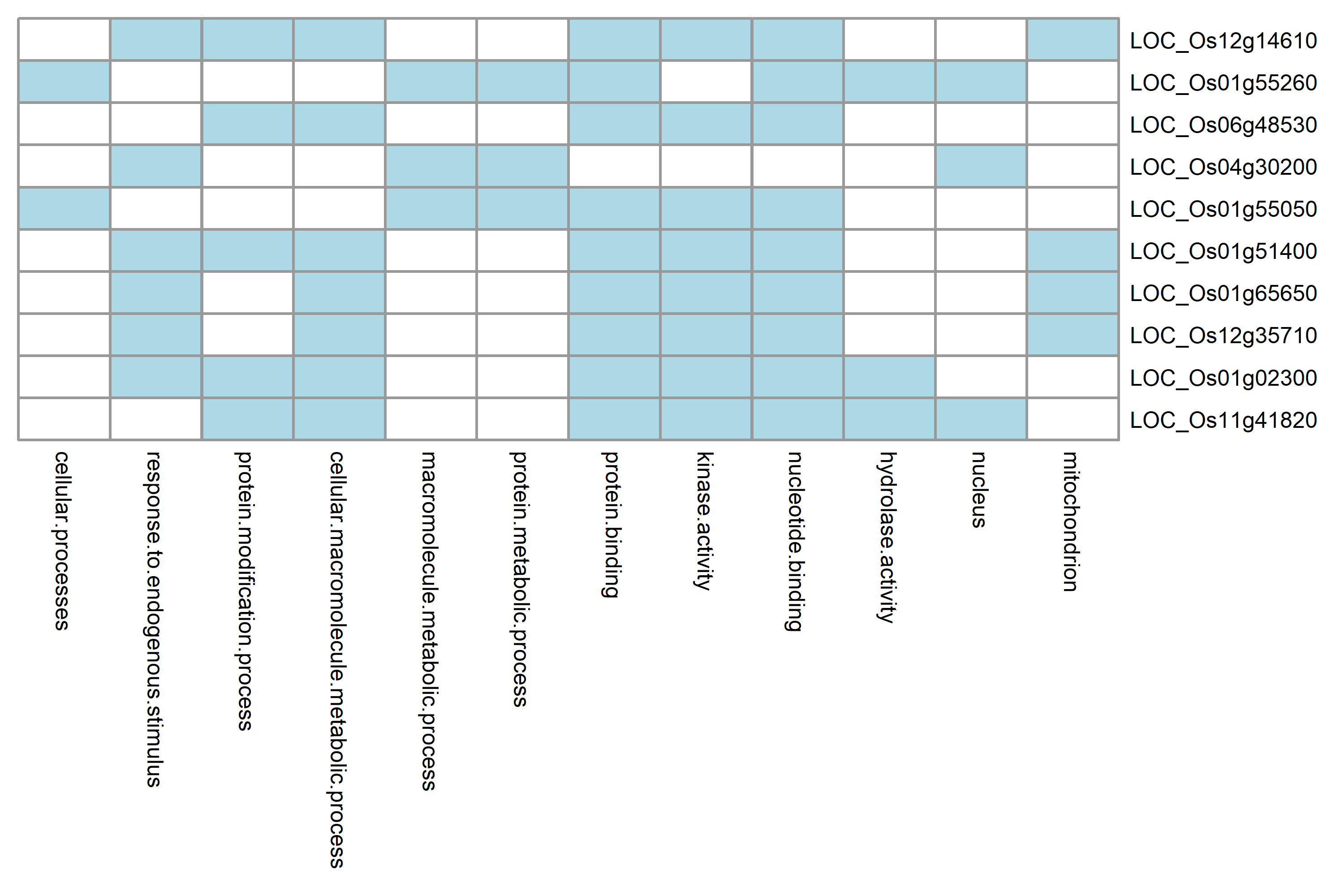

Supplement: Supplementary file 1 [file plants-11-02504-s001.zip › Supplementary Figure S2.tiff]
